# Association of short-term hospital-level outcome metrics with 1-year mortality and recurrence for US Medicare beneficiaries with ischemic stroke

**DOI:** 10.1371/journal.pone.0289790

**Published:** 2023-08-10

**Authors:** Yun Wang, Erica C. Leifheit, Larry B. Goldstein, Judith H. Lichtman

**Affiliations:** 1 Center for Outcomes Research and Evaluation, Yale-New Haven Hospital, New Haven, Connecticut, United States of America; 2 Section of Cardiovascular Medicine, Department of Internal Medicine, Yale School of Medicine, New Haven, Connecticut, United States of America; 3 Department of Chronic Disease Epidemiology, Yale School of Public Health, New Haven, Connecticut, United States of America; 4 University of Kentucky College of Medicine and Kentucky Neuroscience Institute, Lexington, Kentucky, United States of America; The University of Texas MD Anderson Cancer Center, UNITED STATES

## Abstract

**Background:**

Whether stroke patients treated at hospitals with better short-term outcome metrics have better long-term outcomes is unknown. We investigated whether treatment at US hospitals with better 30-day hospital-level stroke outcome metrics was associated with better 1-year outcomes, including reduced mortality and recurrent stroke, for patients after ischemic stroke.

**Methods:**

This cohort study included Medicare fee-for-service beneficiaries aged ≥65 years discharged alive from US hospitals with a principal diagnosis of ischemic stroke from 07/01/2015 to 12/31/2018. We categorized patients by the treating hospital’s performance on the CMS hospital-specific 30-day risk-standardized all-cause mortality and readmission measures for ischemic stroke from 07/01/2012 to 06/30/2015: Low-Low (both CMS mortality and readmission rates for the hospital were <25^th^ percentile of national rates), High-High (both >75^th^ percentile), and Intermediate (all other hospitals). We balanced characteristics between hospital performance categories using stabilized inverse probability weights (IPW) based on patient demographic and clinical factors. We fit Cox models assessing patient risks of 1-year all-cause mortality and ischemic stroke recurrence across hospital performance categories, weighted by the IPW and accounting for competing risks.

**Results:**

There were 595,929 stroke patients (mean age 78.9±8.8 years, 54.4% women) discharged from 2,563 hospitals (134 Low-Low, 2288 Intermediate, 141 High-High). For Low-Low, Intermediate, and High-High hospitals, respectively, 1-year mortality rates were 23.8% (95% confidence interval [CI] 23.3%-24.3%), 25.2% (25.1%-25.3%), and 26.5% (26.1%-26.9%), and recurrence rates were 8.0% (7.6%-8.3%), 7.9% (7.8%-8.0%), and 8.0% (7.7%-8.3%). Compared with patients treated at High-High hospitals, those treated at Low-Low and Intermediate hospitals, respectively, had 15% (hazard ratio 0.85; 95% CI 0.82–0.87) and 9% (0.91; 0.89–0.93) lower risks of 1-year mortality but no difference in recurrence.

**Conclusions:**

Ischemic stroke patients treated at hospitals with better CMS short-term outcome metrics had lower risks of post-discharge 1-year mortality, but similar recurrent stroke rates, compared with patients treated at other hospitals.

## Introduction

Mortality and recurrence are important risks faced by patients discharged after hospitalization for ischemic stroke [1–3]. CMS publicly reports hospital-specific 30-day risk-standardized mortality and readmissions for multiple conditions, including ischemic stroke, and extensive national efforts to improve hospital care have focused on these measures for the past decades [4]. Whether stroke patients treated at better-performing hospitals on one or both of the CMS short-term outcome metrics for stroke (i.e., hospitals with low post-stroke 30-day risk-standardized all-cause mortality and/or low 30-day risk-standardized all-cause readmissions) have lower risks of 1-year post-discharge mortality and recurrent stroke compared to those treated at other hospitals is unknown. The lack of empirical evidence based on contemporary national data represents an important gap in knowledge regarding long-term outcomes for these patients. Such information could inform the development of better care transitions and post-hospitalization secondary prevention efforts to improve long-term outcomes for stroke patients. Accordingly, we used hospital-level CMS 30-day all-cause mortality and readmission performance data for ischemic stroke and patient-level Medicare fee-for-service inpatient claims data to investigate whether publicly available short-term hospital outcome metrics may be an indicator of patient outcomes at 1 year after discharge for ischemic stroke.

## Methods

### Study sample

We used Medicare fee-for-service inpatient claims data to identify beneficiaries who were discharged alive after an initial hospitalization for ischemic stroke from a US acute-care hospital between July 1, 2015 and December 31, 2018. Ischemic stroke was identified using the *International Classification of Diseases*, *Ninth/Tenth Revision*, *Clinical Modification* (ICD-9/10-CM) principal discharge diagnosis code (ICD-9-CM 433, 434, or 436; ICD-10-CM I63). For patients with more than one stroke hospitalization during the study period, we selected the first as the index event. Included patients were required to have at least 12 months free from stroke (2014 inpatient claims data were used to verify this information for patients hospitalized in 2015). We excluded patients who had a length of stay (LOS) ≤1 day (because these patients were unlikely to have had a stroke), those who had conflicting dates of death and hospitalization, those who died at discharge, and those who transferred to another acute-care hospital if the principal discharge diagnosis for the receiving hospital was not ischemic stroke. The Yale University Institutional Review Board approved the study and waived informed consent.

### Hospital short-term outcome performance

We categorized patients according to the performance of the treating hospital on the CMS hospital-specific 30-day risk-standardized all-cause mortality and all-cause readmission measures for ischemic stroke. These short-term hospital outcome metrics are publicly reported and available from the CMS Care Compare website [5, 6]. Data include hospital-specific total discharges and 30-day all-cause risk-standardized mortality and readmission rates for all US acute-care hospitals treating at least 25 Medicare fee-for-service stroke patients aged ≥65 years. The rate measures are based on combined data for 3 years for each hospital and were estimated by CMS using their method for profiling hospitals [7]. Briefly, using a hierarchical generalized linear modeling approach that accounts for patient characteristics and permits hospital-level random intercepts, CMS calculates a risk-standardized ratio, defined as hospital-specific “predicted” deaths divided by hospital-specific “expected” deaths, for each hospital. This ratio, multiplied by the overall national mortality rate, is the risk-standardized mortality rate for an index hospital. A higher-than-expected mortality rate is indicated when the risk-standardized rate is greater than the national rate. The same approach is used to calculate a risk-standardized rate for hospital readmissions.

We used July 1, 2012 to June 30, 2015 as the hospital-level short-term outcome performance measure reporting period to ensure there was no overlap between the performance measure reporting period and our study period for identifying patients discharged with ischemic stroke (i.e., July 1, 2015 to December 31, 2018). Patients discharged from hospitals without data for both the CMS mortality and readmission measures for the July 1, 2012 to June 30, 2015 reporting period were excluded (n = 36,914 patients from 1,756 hospitals). We defined three mutually exclusive categories based on the CMS risk-standardized 30-day mortality and readmission rate measures: 1) Low-Low (both mortality and readmission rates for the treating hospital <25^th^ percentile of the national rates), 2) High-High (both rates >75^th^ percentile of the national rates), and 3) Intermediate (all other hospitals).

### Patient and hospital characteristics

Patient baseline characteristics were obtained from Medicare data for the index stroke hospitalization and included sociodemographic factors (age [continuous], sex [male, female], race [white, black, other], dual eligible for Medicare and Medicaid [8], living in a health priority area [9], living in an area where the median income was <25^th^ percentile of the national level [10], and distance from home to hospital), admission information (weekend hospital admission, admitted from a skilled nursing facility/intermediate care facility, and admitted from an emergency department), clinical comorbidities used in our previous studies [11–16], and history or current smoking. Hospital characteristics were obtained from the American Hospital Association’s Annual Survey Database and included teaching status, Joint Commission certification status, geographic location (urban, rural), ownership (private not-for profit, others), and bed size. Hospital stroke volume was calculated from the Medicare inpatient data.

### Patient outcomes

Our outcomes were all-cause mortality and rehospitalization for ischemic stroke (identified by the ICD-9/10-CM codes described above) within 1 year after the discharge date for the index stroke hospitalization. For patients with more than one rehospitalization for recurrent stroke, the first recurrence was assessed. We also assessed in-hospital major complications (bleeding, acute deep vein thrombosis, pneumonia, pulmonary embolism, septicemia, and infections) based on the secondary diagnosis codes, LOS (continuous), and discharge disposition (home, skilled nursing facility, and inpatient rehabilitation).

### Statistical analysis

We performed descriptive analyses to quantify the differences in patient characteristics, hospital characteristics, and 1-year mortality and recurrent stroke rates across the three hospital short-term outcome performance categories. We evaluated the association between patient risk of dying within 1 year after discharge and hospital performance category using inverse probability weighting (IPW) to reduce potential bias related to differences in patient characteristics between hospital performance categories. Multinomial logistic regression was first used to estimate the conditional probability of a patient to be treated at a Low-Low or Intermediate hospital based on the patient’s baseline sociodemographic and clinical characteristics, distance from home to hospital, and weekend hospital admission. We then used Cox regression, with hospitals as random effects, to model time to death as a function of the hospital performance categories, with treatment at a High-High hospital as the reference, weighted by the inverse of the estimated conditional probabilities and accounting for potential secular trend in the outcome by including an ordinal time variable ranging from 0 (year 2015) to 3 (year 2018). Standard errors were calculated using the robust sandwich approach. We generated adjusted Kaplan-Meier survival curves for mortality according to hospital performance category based on the IPW [17]. We repeated the analysis for the 1-year recurrent stroke outcome, treating patients who switched to a Medicare Advantage plan after the index stroke hospitalization as lost to follow-up and accounting for deaths prior to recurrence as competing risks using the Fine and Gray method [18]. For both outcomes, patients were censored if they did not have the event by 1 year or the end of the study period.

Analyses were conducted using SAS v9.4 (SAS Institute, Cary, NC). The study followed guidelines for cohort studies described in the Strengthening the Reporting of Observational Studies in Epidemiology (STROBE) Statement: Guidelines for Reporting Observational Studies [19]. Statistical tests used a two-sided α of 0.05.

## Results

### Study sample

There were 595,929 unique Medicare beneficiaries discharged with ischemic stroke between July 1, 2015 and December 31, 2018. The mean (SD) age of these patients was 78.9 (8.8) years, and 54.4% were women, 82.2% were White, 11.9% were Black, and 6.0% were of another race. Overall, 0.98% (95% confidence interval [CI] 0.96%-1.01%) of patients switched to Medicare Advantage within 1 year after the index stroke hospitalization.

### Hospital short-term outcome performance categories

**Fig 1:**
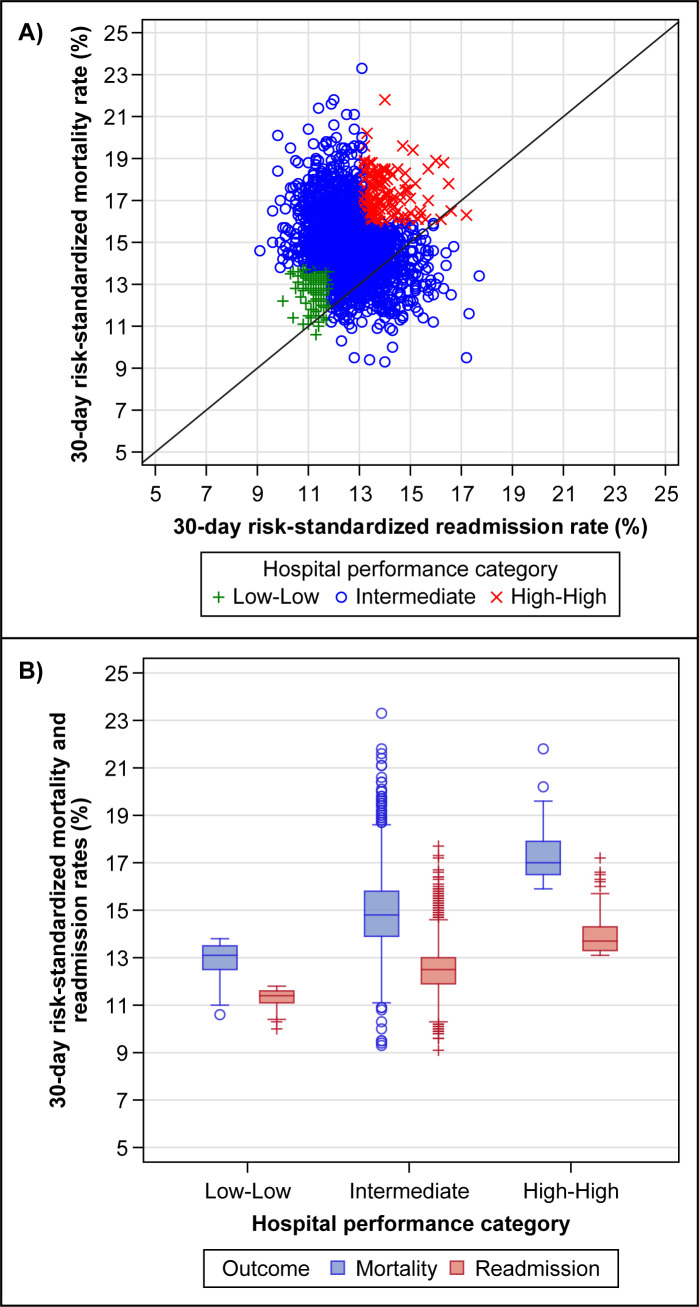
Hospital short-term outcome performance categories. The scatter plot in Panel A shows the Centers for Medicare & Medicaid Services (CMS) 30-day all-cause risk-standardized mortality and readmission rate measures for hospitals in the 2012–2015 reporting period. Each symbol represents a hospital. The box and whisker plots in Panel B show the distributions of the mortality and readmission rate measures by hospital performance category. The box height represents the interquartile range, the horizontal line bisecting the box represents the median value, the whiskers represent the values 1.5 times below the 25^th^ percentile and 1.5 times above the 75^th^ percentile, and the circles and pluses represent outlier hospitals. Low-Low indicates hospitals with both CMS 30-day mortality and readmission rate measures <25^th^ percentile of the national rates. High-High indicates hospitals with both measures >75^th^ percentile of the national rates. Intermediate indicates all other hospitals.

**Fig 2:**
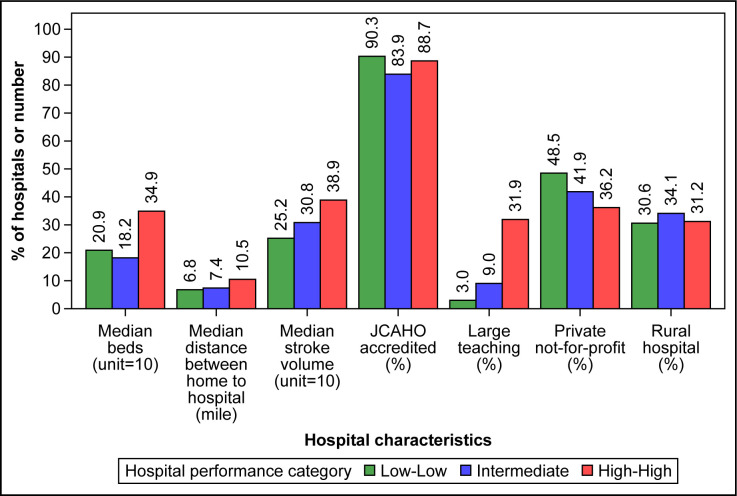
Hospital characteristics by performance category. Hospital short-term outcome performance categories were defined as Low-Low (both Centers for Medicare & Medicaid Services 30-day all-cause risk-standardized mortality and readmission rate measures <25^th^ percentile of the national rates), High-High (both measures >75^th^ percentile of the national rates), and Intermediate (all other hospitals). For Low-Low, Intermediate, and High-High hospitals, respectively, the interquartile ranges were 12.9–29.3, 10.0–31.7, and 22.0–51.2 for median number of beds (unit = 10), 3.0–15.0, 3.0–19.0, and 4.0–34.0 for median distance from home to hospital, and 17.2–38.0, 17.3–49.7, and 25.5–53.1 for median stroke volume (unit = 10). We scaled the values for median beds and stroke volume by dividing by 10 to align with the scales of the other variables in the figure. JCAHO indicates Joint Commission on Accreditation of Healthcare Organizations.

The hospital-level short-term outcome performance data came from 2,563 US acute-care hospitals from July 1, 2012 through June 30, 2015 that had both 30-day all-cause mortality and readmission information. Overall, 134 (5.2%) hospitals were categorized as Low-Low, 2288 (89.3%) as Intermediate, and 141 (5.5%) as High-High (Fig 1A). The overall mean (standard deviation [SD]) rates of risk-standardized 30-day all-cause mortality and readmission were 14.9% (1.7) and 12.6% (1.1), respectively; the rates were 12.9% (0.7) and 11.3% (0.4) for Low-Low hospitals, 14.9% (1.6) and 12.6% (1.0) for Intermediate hospitals, and 17.2% (1.0) and 13.9% (0.8) for High-High hospitals (Fig 1B). Compared with High-High and Intermediate hospitals, Low-Low hospitals had a lower volume of stroke cases and were more likely to be private not-for-profit institutions (Fig 2). Low-Low hospitals were also less likely to be large teaching hospitals. Among the large teaching hospitals, 1.6% were categorized as Low-Low, 80.9% as Intermediate, and 17.6% as High-High.

There were 31,210 patients treated at Low-Low hospitals, 512,850 at Intermediate hospitals, and 51,869 at High-High hospitals. There were no marked differences in patient comorbidities across the three hospital performance categories, but patients treated at Low-Low hospitals were more likely to be White, non-dual Medicare-Medicaid eligible, not living in a health priority area, and not living in a low-income area (Table 1). These differences were reduced after application of the IPW. Patients treated at Low-Low hospitals were less likely to have major complications and had a shorter LOS for the index stroke hospitalization.

**Table 1:** Patient characteristics by hospital performance.

	Hospital Performance Category for CMS 30-Day All-Cause Mortality and Readmission (unweighted)^a^			Hospital Performance Category for CMS 30-Day All-Cause Mortality and Readmission (IPW weighted)^a^^,^^b^		
	Low-Low	Intermediate	High-High	Low-Low	Intermediate	High-High
Number of patients	31210	512850	51869	31092	512196	51698
**Sociodemographic characteristics, %**						
Age, mean (SD), y	79.3 (8.9)	79.0 (8.9)	78.1 (8.7)	78.9 (8.9)	78.9 (8.9)	78.8 (8.7)
Women	54.5	54.6	53.2	54.4	54.4	54.4
Race/ethnicity						
White	85.7	82.3	78.7	82.3	82.2	81.9
Black	7.2	11.7	16.1	9.1	11.8	13.5
Other	7.1	6.0	5.2	8.6	6.0	4.5
Dual Medicare-Medicaid eligible	14.8	16.3	16.8	16.4	16.2	16.3
Residence in area with median income <25^th^ percentile of the national level	6.4	9.2	13.2	9.0	9.5	9.8
Residence in health priority area	17.7	21.8	32.5	21.9	22.6	23.0
Distance from home to hospital, median (IQR), mi	6.8 (3–15)	7.4 (3–19)	10.5 (4–34)	7.0 (3–16)	7.3 (3–20)	8.7 (4–28)
**Comorbid conditions, %**						
Hypertension	58.5	59.3	59.5	59.3	59.3	59.5
Diabetes	33.2	34.5	34.6	34.6	34.4	34.8
History or current smoking	14.3	14.3	14.0	14.2	14.3	14.5
Congestive heart failure	10.0	10.5	10.3	10.5	10.5	10.6
Prior myocardial infarction	2.2	2.2	2.2	2.2	2.2	2.2
Unstable angina	1.5	1.5	1.4	1.5	1.5	1.5
Chronic atherosclerosis	24.4	24.6	23.6	24.6	24.5	24.8
Peripheral vascular disease	6.5	6.7	6.7	6.7	6.7	6.8
Prior stroke	2.2	2.3	2.5	1.2	1.2	1.2
Cerebrovascular disease	28.6	30.6	33.9	30.6	30.8	30.5
Renal failure	23.8	23.1	22.2	23.1	23.1	23.1
Chronic obstructive pulmonary disease	12.5	13.0	12.9	13.0	13.0	13.0
Asthma	3.1	3.1	2.7	3.1	3.0	3.1
Pneumonia	7.0	7.4	7.5	7.4	7.4	7.4
Respiratory failure	5.2	5.1	4.9	5.2	5.1	5.2
Protein-calorie malnutrition	5.9	6.3	6.2	6.3	6.3	6.3
Function disability	3.3	3.5	3.4	3.5	3.5	3.5
Anemia	16.7	17.0	16.5	17.0	16.9	17.0
Parkinson’s or Huntington’s disease	2.3	2.1	2.0	2.1	2.1	2.1
Dementia	18.6	18.7	17.6	18.6	18.6	18.6
Depression	7.8	7.4	7.0	7.4	7.4	7.4
Other psychiatric disorder	2.1	2.1	2.0	2.1	2.1	2.1
Trauma in the past year	5.7	5.9	5.9	6.0	5.9	6.0
Cancer	7.2	6.8	6.6	7.1	6.8	6.6
Liver disease	2.2	2.3	2.3	2.3	2.2	2.3
**Admission information, %**						
Weekend admission	27.2	27.4	27.7	27.4	27.4	27.3
Admitted from ICF/SNF	1.8	2.1	1.8	2.1	2.1	2.2
Admitted from emergency department	84.5	81.9	73.7	81.6	81.3	81.1
Treated at rural hospital^c^	28.8	30.1	29.0	N/A	N/A	N/A
Treated at large teaching hospital^c^	7.0	20.8	48.3	N/A	N/A	N/A
Treated at private, not-for-profit hospital^c^	82.5	77.0	68.7	N/A	N/A	N/A
**Index hospitalization, %**						
Major complication during stay^c^	27.0	28.5	31.3	N/A	N/A	N/A
Length of stay, mean (SD), day^c^	4 (3.8)	4 (4.6)	5 (5.3)	N/A	N/A	N/A
Length of stay >5 day^c^	19.7	22.9	28.4	N/A	N/A	N/A
Discharged to home^c^	31.6	30.2	29.1	N/A	N/A	N/A
Discharged to inpatient rehabilitation^c^	22.2	22.6	25.1	N/A	N/A	N/A
Discharged to ICF/SNF^c^	24.6	25.2	23.5	N/A	N/A	N/A

Abbreviations: CMS, Centers for Medicare & Medicaid Services; ICF, intermediate care facility; IQR, interquartile range; IPW, inverse probability weight; SD, standard deviation; SNF, skilled nursing facility.

^a^Hospital performance categories were defined as Low-Low (both CMS 30-day mortality and readmission rates <25th percentile of the national rates), High-High (both measures >75th percentile of the national rates), and Intermediate (all other hospitals).

^b^Total number of patients for each performance group was based on the inverse probability weights.

^c^Because the IPW approach was used to reduce potential bias related to differences in patients treated at hospitals in the three performance categories, only baseline patient characteristics were included.

### One-year mortality

**Fig 3:**
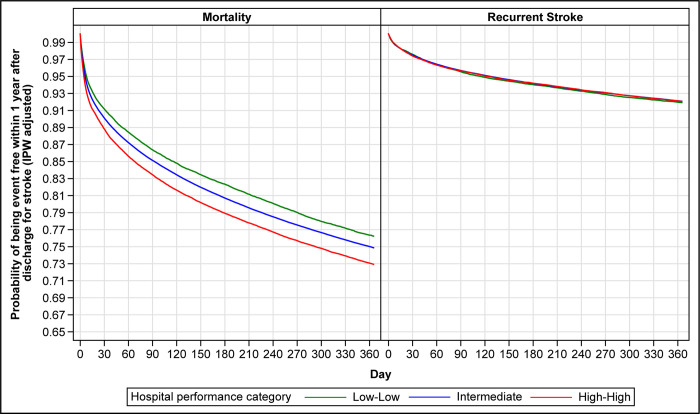
Inverse probability weight-adjusted Kaplan-Meier mortality and recurrent stroke curves. The inverse probability weight-adjusted Kaplan-Meier curves compare 1-year survival for mortality and recurrent stroke for the three hospital performance categories based on the Centers for Medicare & Medicaid Services (CMS) hospital-specific 30-day risk-standardized mortality and readmission measures. Low-Low indicates hospitals with both CMS 30-day mortality and readmission rate measures <25^th^ percentile of the national rates. High-High indicates hospitals with both measures >75^th^ percentile of the national rates. Intermediate indicates all other hospitals. IPW indicates inverse probability weight.

During the 1-year period after the index stroke hospitalization, 25.2% (95% CI 25.1%-25.4%) of patients died. There were marked differences in 1-year mortality across the hospital short-term outcome performance categories. Mortality was 23.8% (95% CI 23.3%-24.3%) among patients treated at Low-Low hospitals, 25.2% (95% CI 25.1%-25.3%) among patients at Intermediate hospitals, and 26.5% (95% CI 26.1%-26.9%) among patients at High-High hospitals. These rates did not change substantially after adjusting for potential differences in patient baseline characteristics across the performance categories (Fig 3, left panel). Accounting for IPW, 1-year mortality was lower for patients initially treated at Low-Low (hazard ratio [HR] 0.85; 95% CI 0.82–0.87) and Intermediate (HR 0.91; 95% CI 0.89–0.93) hospitals compared with patients treated at High-High hospitals.

### One-year stroke recurrence

The observed 1-year recurrent stroke rate was 7.9% (95% CI 7.8%-8.0%). There were no differences in 1-year recurrence across the hospital short-term outcome performance categories. For Low-Low, Intermediate, and High-High hospitals, the observed recurrence rates were 8.0% (95% CI 7.6%-8.3%), 7.9% (95% CI 7.8%-8.0%), and 8.0% (95% CI 7.7%-8.3%), respectively. These rates did not change substantially after adjusting for potential differences in patient baseline characteristics across the performance categories (Fig 3, right panel). The IPW-adjusted HRs were 1.05 (95% CI 0.99–1.11) for patients treated at Low-Low hospitals and 1.01 (95% CI 0.98–1.05) for patients treated at Intermediate hospitals, compared with patients treated at High-High hospitals.

## Discussion

In this analysis using nationwide data for Medicare fee-for-service beneficiaries, we found that ischemic stroke patients aged ≥65 years who were discharged from US hospitals with better performance on the publicly reported CMS short-term hospital mortality and readmission metrics had lower risks of 1-year mortality but not recurrent stroke when compared with patients treated at other hospitals. The differences in mortality by hospital short-term outcome performance category were apparent in the early period after hospital discharge following the index stroke and persisted throughout the 1-year follow-up period.

Our study represents a novel approach for combining publicly reported hospital-level short-term outcome performance measures with stroke patient-level data to assess the association between short-term hospital performance metrics and longer-term patient outcomes. We observed differences in post-stroke discharge mortality rates by hospital performance category, which may reflect differences in the organization, quality, or receipt of care provided. Patients who were treated at Low-Low hospitals were less likely to have a major complication and had a shorter LOS, which are associated with better shorter-term outcomes [20–22]. These findings may reflect aspects of hospital care, such as patient safety culture, discharge planning, and care transitions, which are increasingly recognized as important for condition-wide hospital outcomes [23–26]. There was a mix of hospital characteristics across the performance categories, indicating opportunities for improvement by characteristics including hospital size, stroke volume, and teaching status. Approximately 3% of Low-Low hospitals, 9% of intermediate hospitals, and 31.9% of High-High hospitals were large teaching institutions. Although a larger proportion of High-High hospitals were large teaching institutions, there were teaching hospitals categorized as Low-Low, suggesting that even large teaching institutions that typically treat patients with more complex illnesses can have among the best short-term outcomes as reflected by short-term hospital performance metrics [27].

We observed marked differences in patient demographic characteristics, complications, and LOS, but not patient comorbidities, across the hospital short-term outcome performance categories. We found that patients who were Black, dual-eligible for both Medicare and Medicaid, and living in a low-income or health priority area were more likely to be treated by hospitals that performed poorly on the CMS 30-day measures. These findings highlight the importance of continuing national efforts to reduce disparities in health care across populations. Optimization of post-discharge care for these patients may be particularly important for reducing post-discharge mortality.

Our findings may reflect differences in post-discharge care, secondary prevention, and quality of care at the community level across hospital performance categories [28–31]. Community-level efforts involve an integrated approach with hospitals, primary care providers, rehabilitation facilities, nursing homes, and home health agencies partnering together to ensure patients receive optimal post-discharge care across venues and care providers. The index hospital alone may lack the resources to achieve this integrated approach, and national interventions may be needed to improve post-discharge care. The opportunity to identify patients at risk for adverse long-term outcomes can inform targeted hospital-level and community-level interventions to optimize care transitions and post-hospitalization secondary prevention efforts for stroke patients who may require additional attention for follow-up care.

Our study has limitations. Because our study focused on longer-term poststroke mortality and recurrence, we limited our sample to patients discharged alive after stroke. This could introduce some bias as those with severe stroke leading to in-hospital death related to differences in hospital performance were excluded. Our recurrence outcome included only strokes resulting in a re-hospitalization and did not include recurrences that may have occurred outside of the hospital setting. We focused on the first hospitalization for a recurrent stroke and did not account for multiple recurrent events. Information on medication adherence, nursing home stays, and home health services were not included in our data but may be associated with outcomes. Comorbidities were identified from billing codes, and administrative data lack detailed clinical information on patient functional status and severity of the index stroke. Nevertheless, this study distinguishes itself by the breadth and standardization of the outcomes measured and its national scope.

## Conclusion

Ischemic stroke patients treated at hospitals with better short-term hospital outcome metrics for stroke had a lower risk of post-discharge 1-year mortality, but similar recurrent stroke rates, compared with patients treated at other hospitals. The results underscore the importance of using national data to conduct outcome surveillance beyond the early recovery period, and they highlight the potential of using existing metrics to identify patterns of outcomes that represent opportunities to improve long-term care for stroke patients.
